# Automatic Daily Activity Schedule Planning for Simulating Smart House with Elderly People Living Alone

**DOI:** 10.1007/978-3-030-51517-1_14

**Published:** 2020-05-31

**Authors:** Can Jiang, Akira Mita

**Affiliations:** 8 Digital Research Centre of Sfax, Sfax, Tunisia; 9 Institut Mines-Télécom, CNRS, Paris, France; 10 Université de Sherbrooke, Sherbrooke, QC Canada; 11 Digital Research Centre of Sfax, Sfax, Tunisia; 12 University of Sfax, Sfax, Tunisia; 13 Graduate School of Science and Technology, Keio University, 3-14-1 Hiyoshi, Kohoku-ku, Yokohama, Kanagawa Japan; 14 Department of System Design Engineering, Keio University, 3-14-1 Hiyoshi, Kohoku-ku, Yokohama, Kanagawa Japan

**Keywords:** Elderly people living alone, Smart home simulator, Activity of daily living, Motivation, Automatic scenario generation

## Abstract

A simulation tool that supports developers to build scenarios automatically in multiple simulation platforms is proposed. As an essential part of this simulator, this study proposed an activity schedule generator to mimic the daily life of elderly people living alone. This generator outperforms existing methods of activity schedule planning in three aspects: 1) it is adaptive to the layout of a simulated smart house; 2) there is no unspecified time in the timeline of generated schedules; and 3) it generates stable, but not tedious schedules for a number of days. A real-time location data generator is proposed to convert generated schedules to simulated real-time location data of the resident, and a proposed interface converts these simulated location data to simulated records of virtual passive infrared (PIR) sensors, which can be used to optimize placement of PIR sensors in a smart house.

## Introduction

The elderly population is increasing worldwide. An estimated 617.1 million people are aged 65 and over in 2015, and this number is projected to increase to 1 billion in 2030, and 1.6 billion in 2050 [1]. More than 20% of men and 40% of women aged 65 and older chose an independent lifestyle in many countries [2]. Pimouguet et al. [3] indicated living alone shortened life expectancy by 0.6 years for elderly people. Elderly individuals living alone would benefit from specialized care, but a shortage in the global workforce of aged-care workers [4] has made this difficult.

Under these conditions, smart houses with a sensor network and domestic robots have been built to address the aged-care worker shortage. The sensor networks provide real-time health monitoring [5] and a means of detecting emergencies [6], while mobile domestic robots provide location-based support [7] and services [8] for residents. To ensure the effectiveness of the sensor networks and robots, real test beds were built to conduct experiments for collecting data. However, building a test bed is expensive, and simulations are necessary for smart house developers to test and verify their ideas before building a real one.

Developers typically conduct simulations using the following three steps. (1) Manually create a simulation scenario by first building a house and resident body models and defining the activity schedules and movement routes of the virtual resident or controlling the virtual resident manually. (2) Place virtual sensors, devices, or robots to record data and/or operation performances. (3) Analyze recorded data or operation performances and evaluate simulation design. As a typical simulation constructed in step (1) requires a lot of time, developers can only prepare a limited number of scenarios. Moreover, the developers may use multiple simulators for different purposes, e.g., using CST Microwave Studio to test the communication of a wireless sensor network, OpenSHS [9] to collect virtual sensor records for sensor arrangement optimization, and Stage [10] to plan the operation policies of mobile robots. When the developers use another simulator, they must repeat steps (1) even if they use the same simulation scenario.

**Fig. 1.**
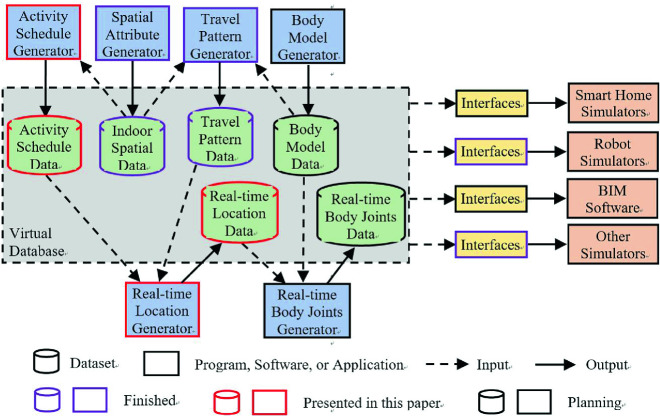
General framework for building the simulation tool [11].

We propose a simulation tool that provides diverse simulation scenarios and can support smart house developers to complete step (1) automatically in multiple simulation platforms [11]. This simulator consists of generators and interfaces as show in Fig. 1. The proposed generators produce diverse information such as indoor spatial attributes and resident travel patterns. This information is used to create a scenario that can run on different simulation platforms through various interfaces. We proposed a spatial attribute generator [12] and travel pattern generator [11], and used two interfaces [11] to transfer the data generated by them to models and virtual sensor records of the simulators.

As an essential part of our simulator, we propose an activity schedule generator. With generated travel patterns, these schedules are converted to simulated real-time location data, which can be used in simulations with interfaces. The rest of this paper is organized as follows. In Sect. 2, we review related work of daily activity schedule generation. Section 3 describes the methodology to generate activity schedules. Section 4 details the performance of this generator. Section 5 introduces how the generated data can be used in simulations.

## Related Works

A number of scholars generated daily activity schedules as intermediate results to generate sensor records in a virtual smart house, which are essential for simulations.

Renoux et al. [13] generated activity schedules with a constraint-based planning method. The constraints include that the start time and duration of each activity are over reasonable intervals, and a number of activities need to be performed within certain time intervals before their corresponding activities, e.g., preparing lunch for 0 to 5 min before having lunch.

Bouchard et al. [14] generated activity schedules using behavior trees (BTs) as intermediate results to generate the simulated evolution of signal strength between RFID readers and tags. However, designing BTs is complicate, and the authors only showed an example of generating the schedule for making coffee or tea.

Alshammari et al. [9] replicated and modified schedules originally designed by humans. The methods of modification include combining two samples of original schedules and changing the start and end time of activities. The activity schedules correspond to virtual binary sensor records, thus, a large number of records are generated simultaneously. This method is simple, but generated schedules may have high similarity.

Mshali et al. [15] generated long-term activity schedules using a Markov model, and five transition matrices associated to different periods of a day were designed. The authors also proposed an adaptive and context-aware algorithm for monitoring the daily activities of elderly and dependent persons, and generated schedules were used to test the algorithm in simulations.

Lee et al. [16] generated activity schedules with a motivation-driven method. A motivation value (MV) represents the desire of a virtual agent to perform a class of activities with the agent performing an activity when its corresponding MV reaches its threshold. Motivations are classified by levels; if two MVs reach their thresholds at the same time, the agent will perform the activity that corresponds to the higher-level motivation. This method has sufficient potential for improvement if the mechanism of evolution of the MVs is designed carefully.

## Activity Schedule Generation

### Problem Statement

To build our activity schedule generator module, we need to improve upon the methods mentioned in Sect. 2 by addressing the following issues. 1) The list of activities that can be performed by a virtual resident is determined by the layout of simulated house, e.g., the resident can only watch TV if a TV is in the house. The above methods are for a determined layout with a fixed activity list. As our simulator contributes to provide diverse simulation scenarios by producing diverse layouts, we need a method that can process dynamic activity lists. 2) The above methods generate schedules whose timelines include unspecified times between the end time of an activity and the start time of the next one. Where the resident has been and what he/she has done during the unspecified time are undetermined, thus, generated sensors records did not cover entire days. 3) Most of the above methods generated schedules for one day or less, but long-term activity schedules are required for our simulation.

We developed a motivation-driven method on the basis of that presented in the reviewed study [16] to build our activity schedule generator. An MV represent a resident’s desire to perform an activity sequence (AS). While performing the activity is dependent on its MV reaching its threshold in [16], in our method, the MVs are used to determine the probability distribution (***P***) of sampling the next AS. The evolution of the MVs is adaptive to the input indoor spatial data and resident’s profile. The input data represent a layout that determines what AS can be performed, thus, this adaptive evolution mechanism addresses issue 1). The profile represents a resident’s tendencies to activities, which is quantified by durations (***D***), periods (***T***) and frequencies (***f***) of an AS. We need to design an evolution mechanism and initialize the MVs carefully to generate stable, but not tedious, activity schedules in the long term that will address issue 3). We can address issue 2) by taking into account more activities. The studies mentioned in Sect. 2 took into account a limited number of activities, implying that these activities occupy the entire timeline, which is unrealistic.

### Model of AS, *MV*, Resident Profile, and *P*

#### Mapping the Relationship Between AS and *MV*.

A resident performs activities on the basis of motivation, in which MVs quantify the degree of motivation. When an MV is high, the resident may perform a sequence of activities to satisfy the motivation, e.g., if the value of hunger motivation is high, he/she will cook and eat.

We determined that a resident has 13 motivations at most, which correspond to 13 *MV*_i_-*AS*_i_ pairs: *MV*_1_: wash and brush teeth => sleep (at night) => wash and brush teeth, *MV*_2_: sleep (at noon), *MV*_3_: take food => cook => take tableware => eat, *MV*_4_: take a bath => get dressed => put clothes in wash machine, *MV*_5_: get dressed => go out => get dressed, *MV*_6_: go to toilet (short duration), *MV*_7_: go to toilet (long duration), *MV*_8_: watch TV, *MV*_9_: read, *MV*_10_: clean, *MV*_11_: take clothes out of washing machine, *MV*_12_: wander, and *MV*_13_: relax. An AS consists of one or more activities, and activities in the AS are performed in order without lag to satisfy the corresponding motivation and decrease the corresponding *MV*_*i*_. *MV*_*i*_ determines the possibility of performing *i*th AS, *P*_*i*_.

#### AS and *MV* Are Adaptive to the Layout.

The actual composition of an AS is also adaptive to the layout like the evolution of MVs. An activity in an AS will be omitted if its corresponding places are not in the layouts The mapping relationship of all activities and places is shown in Table 1, e.g., if the kitchen stove, refrigerator, and cupboard are not in the house, AS_3_ will omit the procedure take food => cook => take tableware, which implies the resident eats food prepared by someone out of the house in this case. If all activities of an AS can not be performed because of the layout, its *MV*_*i*_ will always be 0.

**Table 1.** Mapping relationship between activities and places.

Activity	Place
Sleep (at night) Sleep (at noon) Relax	Bed
Wash and brush teeth Take a bath	Bathroom
Take food	Refrigerator
Take tableware	Cupboard
Take food Take tableware Cook	Kitchen stove
Eat	Dining table-chair set
Put clothes in washing machine Take clothes out of washing machine	Washing machine
Go to toilet (short duration) Got to toilet (long duration)	Toilet
Watch TV Relax	Sofa-TV set
Relax Read	Writing desk-chair set
Get dressed	Wardrobe
Eat Go out	Entrance
Clean	Trash bin
Wander	None

#### Resident’s Personal Profile.

To keep generated schedules diverse and reasonable, parameters corresponding to the evolution of MVs should depend on the resident’s profile. The profile represents a resident’s tendencies to satisfy different motivations, which is determined by sampling ***D***, ***T***, and ***f*** of an AS performed over reasonable intervals. *Di* means the duration of the resident performing the *i*th AS in a period (*T*_*i*_), e.g., *D*_8_ means how long the resident performed the activity “watch TV” per day on average if *T*_8_ = 1 day. *T*_*i*_ and *f*_*i*_ mean the period and frequency of performing the *i*th AS, respectively, e.g., *T*_12_ means how many days between two instances of the resident’s wandering and *f*_6_ means how many times the resident performed the activity “go to toilet (short duration)” per day on average, where *T*_*i*_ × *f*_*i*_ = 1.

#### Mechanism of MVs Evolution.

MVs quantify the motivations to perform activities, *MV*_*i*_ usually decreases when the *i*th AS is performed, increases when other ASs are performed, and remains unchanged in special cases. The values of ***D***, ***T***, and ***f*** determine the speed of the increase and decrease of MVs. The rules below show the evolution of *MV*s, where *Rules* 1.1) and 1.2) indicate the situations when *MV*_*i*_ remains unchanged, 2.1) to 2.4) show the mechanism of MVs increasing, and 3.1) to 3.4) show the mechanism of them decreasing.

- *Rule 1.1) MV*_*i*_ is always 0 if *i*th AS can not be performed.
- *Rule 1.2) MV*_13_ = 1.02Norm_MV in any case, where Norm_MV is a constant.

Note: *MV*_13_ corresponds to relax. This rule means the resident is performing the activity “relax” when all other MVs are low. Setting *MV*_13_ as a constant keeps MVs stable in the long term.

- *Rule 2.1)* Ways of MVs increasing include linear and step functional increasing.
- *Rule 2.2)* If *MV*_*i*_ is not fixed and *i* ≠ 7 or 11, *MV*_*i*_ increases linearly when the *i*th AS is not performed. The increment is determined by Eq. (1),
  1 $$ MV_{i} \left( {t\, + \,\Delta t} \right) = \left\{ {\begin{array}{*{20}c} {MV_{i} \left( t \right)\, + \,\omega_{i} \Delta t\, + \,\varepsilon , {\text{ if the resident is not sleeping.}}} \\ {MV_{i} \left( t \right)\, + \,0.1\omega_{i} \Delta t\, + \,\varepsilon , {\text{ if the resident is sleeping, }}and \, i = 3{\text{ or }}6.} \\ {MV_{i} \left( t \right)\, + \,\varepsilon , {\text{ if the resident is sleeping, and }}i \ne 3{\text{ and }}6.} \\ \end{array} } \right. $$
  where *ω*_*i*_ is an increasing rate and *ɛ* is random noise.

Note: When the resident is sleeping, the increasing rates of the MVs of “eat” and “go to toilet (short duration)” decrease to 10%, rates of other MVs decrease to 0.

- *Rule 2.3)* Following the principle that *MV*_*i*_ should be generally unchanged after one period in an ideal case, *ω*_*i*_ can be determined by ***D***, ***T***, and ***f***.

Note: e.g., for the 8^th^ AS, watching TV, assuming that the resident watches TV for 4 h (*D*_8_), and sleeps 8 h (*D*_1_ + *D*_2_) per day (*T*_8_ = 24 h), *ω*_8_ is determined by Eq. (2),

2 $$ \omega_{ 8} { = }\frac{{{\text{Norm}}{\_}{\text{MV}}}}{{[T_{8} - (D_{1} + D_{2} ) - D_{8} ]}} . $$

which means *ω*_8_ should increase by Norm_MV in the remaining 12 h, while it decreases by Norm_MV during the 4 h (*D*_8_).

- *Rule 2.4) MV*_*i*_ increases step by step if *i* = 7 or 11. For the 7^th^ AS, “going to toilet (long duration)”, *MV*_7_ increases by Norm_MV/(3 × *T*_7_) 2.5 h after the resident starts eating. For the 11^th^ AS, “taking clothes out of the washing machine”, *MV*_11_ increases by Norm_MV 1 h after the resident puts their clothe into the washing machine.
- *Rule 3.1) MV*_*i*_ will decrease if the *i*th AS has been performed except if *i* = 13.
- *Rule 3.2)* The decrease of *MV*_*i*_ depends on the *T*_*i*_ and actual duration, *AT*. It is defined by Eq. (3) except if the AS is eating breakfast, the decrease is (2*AT/*3*T*_*i*_) × Norm_MV.
  3 $$ MV_{i} \left( {t + AT} \right) = MV_{i} \left( t \right) - \frac{AT}{{T_{i} }}{\text{Norm}}{\_}{\text{MV}} . $$
- *Rule 3.3) AT* is related to *T*_*i*_, *AT* is sampled from [0.97*T*_*i*_, 1.03*T*_*i*_] for *i* = 1, from [0.4*T*_*i*_, 0.9*T*_*i*_] for *i* = 5, from [0.3*T*_*i*_, 0.7*T*_*i*_] for *i* = 8 or 9, and from [0.95*T*_*i*_, 1.05*T*_*i*_] for other cases.
- *Rule 3.4)* The resident may perform the activities “eat” and “go to toilet” outside. When he/she is going out, if *MV*_3_, *MV*_6_ or *MV*_7_ reach Norm_MV, and there is sufficient time to perform the corresponding AS, this MV decreases as the AS is performed.

#### Initialization of MVs.

MVs should be initialized before evolution, which can be achieved by determining when each AS will be performed for first time. The time when the *i*th AS is first performed is approximately equal to the time when *MV*_*i*_ first reaches Norm_MV. We sample the initial time from 9:30 PM of one day to 1:00 AM of the next day, and the resident is going to sleep. *MV*_0_ thus is Norm_MV, as *D*_*i*_ and *ω*_*i*_ is known, other initial MVs can be calculated with Eq. (1), e.g., assuming that *D*_1_ = 8 h, and the resident will eat breakfast 1 h after waking up, initial *MV*_3_ is calculated by Eq. (4).

4 $$ MV_{3} = {\text{ Norm}}{\_}{\text{MV}} \, {-} \, 0.1\omega_{3} \times 8\,{\text{h }}\,{-}\,\omega_{3} \times 1\,{\text{h}}. $$

To keep the generated schedule stable in the long term, we need to avoid two MVs whose ASs require long durations to reach Norm_MV at the same time.

#### Relationship Between MV and P.

The possibility of performing the *i*th AS depends on the motivation value, *MV*_*i*_, as shown in Eq. (5)

5 $$ P_{i} = \frac{{\exp [\hbox{max} (0,MV_{i} - 0.98{\text{Norm}}{\_}{\text{MV}})]}}{{\sum\limits_{j = 1}^{13} {\exp [\hbox{max} (0,MV_{j} - 0.98{\text{Norm}}{\_}{\text{MV}})]} }} . $$

### Implementation

We wrote a Python3 program to achieve activity schedule generation. A sample of the indoor spatial attribute data (***Spatial_data***) and total generation duration (Total_Dur) were input into the program, and it returns a resident’s daily activity schedule during the Total_Dur. The pseudocode of the program is shown below, where constants, variables, and variable vectors are in regular, italic, and bold italic styles, respectively.


program Schedule_Generation(***Spatial_data***, Total_Dur):1***AS_canbe_perform***:= Process_Input(***Spatial_data***)2***T****,****D****,****f***:= Generate_Resident_Profile()3***ω***:= Calculate_Increse_rate(***T****,****D****,****f***)4***MV***, Init_time := Initialize_MVs&time(***T****,****D****,****f***)5*Current_ASnum*,*time*:= 1, Init_time6***ASnum_list****,****time_list***:= [], []7 while*time*< Init_time + Total_Dur do:8*    AT*:= Determine_actual_duration(*Current_ASnum*,***T***)9***    MV***:= Update_MV(*Current_ASnum*,*time*,***MV***,***ω***,**T**,*AT*)10*   time*:=*time*+*AT*11*   Next*_ASnum = Sample_next_ActSeq(***MV***)12    If*Next_ASnum*!=*Current_ASnum*do:13***      ASnum_list***.append(*Next_ASnum*)14***      time_list***.append(*time*)15*      Current_ASnum := Next_ASnum*16 Activity_*Schedule*= Post_Process(***ASnum_list***,***time_list***,***Spatial_data***)17 return Activity_*Schedule*


The program first processes the input spatial attribute data, analyzes the layout, and determines what ASs can be performed in the house in Line 1. The resident’s profile is determined by sampling ***D***, ***T***, and ***f*** in Line 2. ***ω*** is calculated in Line 3 in accordance with *Rule 2.3)*. The original ***MV*** and the start time of the schedule generation are determined in Line 4. In Line 5, we assume the resident performs AS_1_ at the beginning of the generation, and the variable *Time* records the current time. Two lists are created in Line 6, ***ASnum_list*** and ***time_list***, which will record the number of all performed ASs and their start times chronologically, respectively. From Lines 7 to 15, the program determines the *AT* of performing each AS with *Rule 3.3)*, updates ***MV*** using the other rules, samples the next performed AS with Eq. (5), and stores the number of performed ASs and their start times in ***ASnum_list*** and ***time_list***, respectively. The program converts these two lists into an activity schedule in Line 17. The schedule indicates the start times of all activities performed.

## Performance of the Generator

**Fig. 2.**
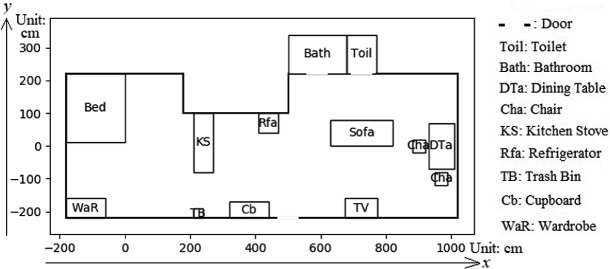
Layout of input indoor spatial data.

**Fig. 3.**
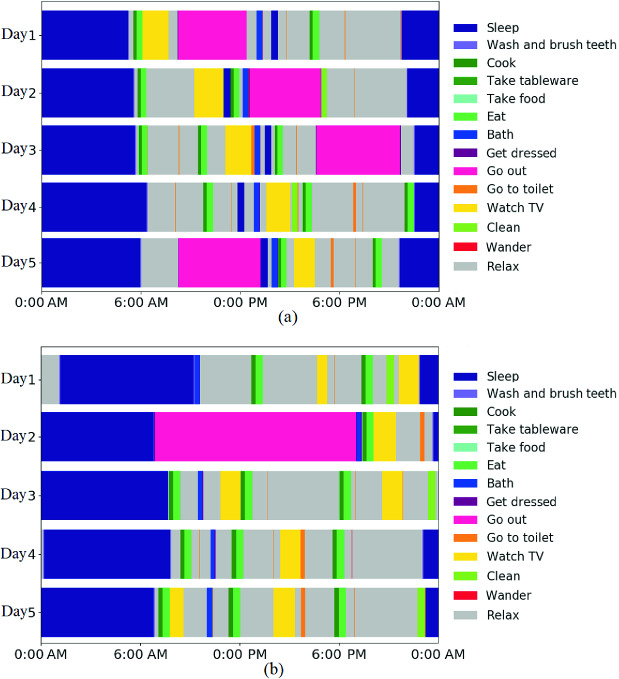
Generated activity schedules.

We input indoor spatial data generated by the spatial attribute generator into the activity schedule generator, which then produces diverse activity schedule data. For example, a sample of spatial data whose layout is shown in Fig. 2 is input into the generator. As the places “desk” and “washing machine” do not exist in the house, AS_9_ (read) and AS_11_ (take clothes) can not be performed. The activity schedule generator then determines the resident’s profiles and generates their corresponding schedules. Two example schedules are shown in Fig. 3. Figure 3a) shows a schedule for a resident who sleeps around noon, goes out, watches TV, and takes a bath every day, while Fig. 3b) shows a schedule for who does not sleep around noon, watches TV, and takes a bath every day, but only goes out every four days.

We also tested the performance of our generator on PC with an Intel^(R)^ core^(TM)^ i7-8550U @1.80-GHz CPU. The generator ran 100 times in 3.987 s.

Additional generated schedules are available via this website [17].

## Using Generated Activity Schedules for Simulation

Smart house are often equipped with passive infrared (PIR) sensors. When residents are in the detection range of one, it turns on, otherwise, it remains off. Each PIR sensor has a unique ID number which can be recorded when the sensor turns on or off. By placing several PIR sensors in the house and analyzing their records, a resident’s movement trajectories can be acquired, which can be used to determine whether they contain wandering travel patterns associated with dementia [18].

In the simulation, the PIR sensor records were generated from simulated real-time location data. We built a generator that could convert an activity schedule, a sample of indoor spatial data, and several samples of travel pattern data into a sample of real-time location data. We developed an interface to convert the real-time location data into virtual PIR sensor records, which can be used to optimize the placement of the PIR sensors in a smart house.

**Fig. 4.**
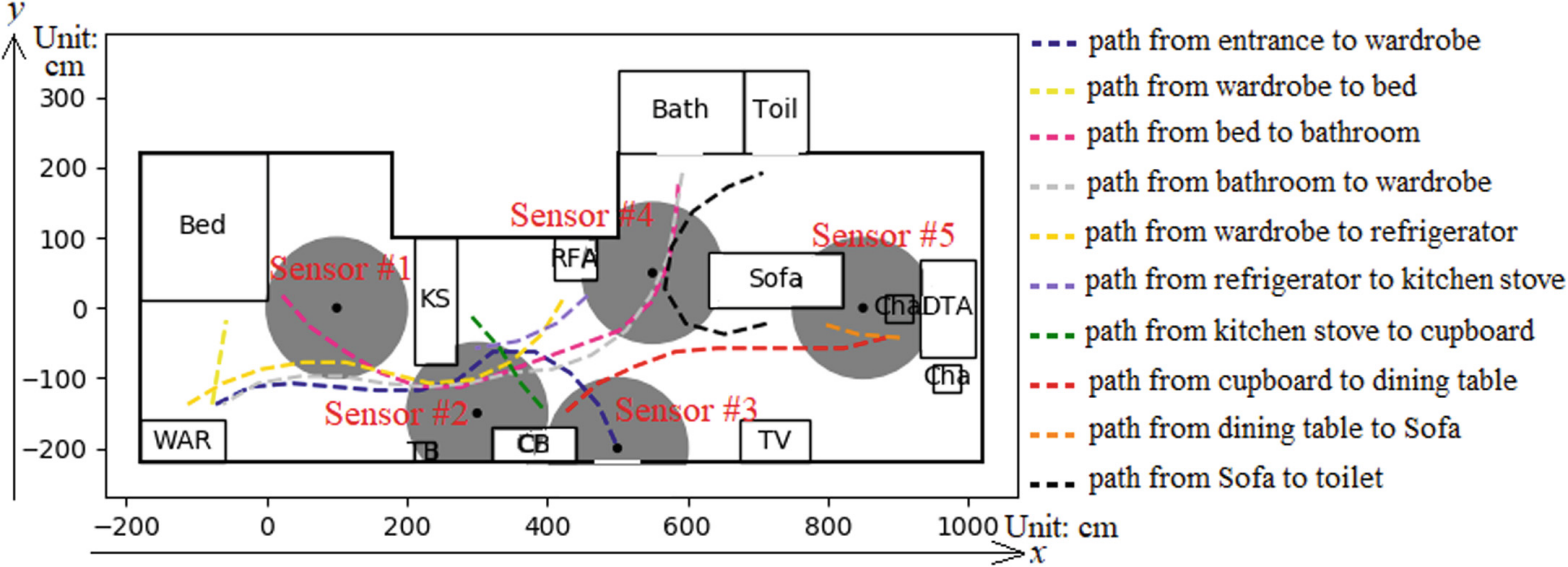
Layout of input indoor spatial data with PIR sensors and travel pattern data.

Examples of the performance of the real-time location data generator and the interface are shown in figures and tables. Figure 2 shows a sample of spatial data. Table 2 shows part of an activity schedule. The travel pattern data are shown in Fig. 4. The above data are input into the generator to produce the real-time location data. Figure 4 also shows the positions of the five PIR sensors located in the virtual house. Their coordinates are [100, 0], [300, −150], [500, −200], [550, 50] and [850, 0]. The interface converts the real-time location data into the records of PIR sensors, which is shown in Table 3.

**Table 2.** Part of the activity schedule shown in Fig. 3(a).

Activity	Start time	Activity	Start time
Go out Get dressed Sleep Relax Bath Get dressed Take food	5d AM 8 h 17 m 7 s 5d PM 1 h 13 m 59 s 5d PM 1 h 16 m 12 s 5d PM 1 h 40 m 0 s 5d PM 1 h 54 m 36 s 5d PM 2 h 15 m 10 s 5d PM 2 h 17 m 18 s	Cook Take tableware Eat Relax Watch TV Go to toilet	5d PM 2 h 17 m 56 s 5d PM 2 h 28 m 44 s 5d PM 2 h 29 m 46 s 5d PM 2 h 49 m 20 s 5d PM 3 h 15 m 1 s 5d PM 4 h 30 m 33 s

**Table 3.** Simulated records of virtual PIR sensors.

Time		Time	
5d PM 1 h 13 m 45 s 9 5d PM 1 h 13 m 47 s 8 5d PM 1 h 13 m 50 s 2 5d PM 1 h 13 m 53 s 4 5d PM 1 h 54 m 19 s 5 5d PM 1 h 54 m 22 s 9 5d PM 1 h 54 m 24 s 2 5d PM 1 h 54 m 27 s 5 5d PM 1 h 54 m 30 s 2 5d PM 1 h 54 m 34 s 4 5d PM 2 h 14 m 52 s 3 5d PM 2 h 14 m 56 s 4 5d PM 2 h 14 m 59 s 1 5d PM 2 h 15 m 2 s 5 5d PM 2 h 15 m 4 s 4 5d PM 2 h 15 m 5 s 1	#3 ON #3 OFF #2 ON #2 OFF #1 ON #1 OFF #2 ON #2 OFF #4 ON #4 OFF #4 ON #4 OFF #2 ON #2 OFF #1 ON #1 OFF	5d PM 2 h 17 m 7 s 5 5d PM 2 h 17 m 9 s 7 5d PM 2 h 17 m 10 s 9 5d PM 2 h 17 m 14 s 0 5d PM 2 h 17 m 50 s 5 5d PM 2 h 17 m 50 s 6 5d PM 2 h 17 m 53 s 6 5d PM 2 h 28 m 38 s 5 5d PM 2 h 28 m 39 s 7 5d PM 2 h 29 m 34 s 5 5d PM 2 h 29 m 34 s 5 5d PM 2 h 29 m 36 s 1 5d PM 2 h 29 m 41 s 9 5d PM 3 h 30 m 21 s 7 5d PM 3 h 30 m 23 s 8 5d PM 3 h 30 m 27 s 7	#1 ON #1 OFF #2 ON #2 OFF #4 ON #4 OFF #2 ON #2 OFF #2 ON #2 OFF #3 ON #3 OFF #5 ON #5 OFF #4 ON #4 OFF

## Conclusion

Smart houses with a sensor network and domestic robots were built to take care elderly people living alone. Many simulation tools have been proposed to help smart house developers test and verify their designs, but it takes time and effort to build a simulation scenario, and developers need to repeat scenario-generation procedures if they want to use multiple simulators. To address these issues, we proposed a simulation tool that provides diverse simulation scenarios and enables developers to build scenarios automatically in multiple simulation platforms [11].

In this paper, we proposed an activity schedule generator that is an essential part of our simulator. With an improved motivation-driven method, the generator produces diverse daily activity schedules to mimic the daily lives of residents living alone. It outperforms existing generators in three aspects: 1) it is adaptive to the layout of a simulated smart house; 2) there is no unspecified time in the timeline of generated schedules; and 3) it generates stable, but not tedious schedules for a number of days.

A generated schedule includes a list of activities and their start time. The list of activities determines all starts and ends of indoor walking paths with spatial attributes of a virtual house, the travel pattern generator then generates all paths. The generated paths determine simulated real-time locations of a resident with the list of start time.

The real-time locations can be converted to records of virtual sensors with interfaces, and these records can be used to optimize designs of smart house. For example, we convert the real-time locations to records of virtual PIR sensors, and the records are useful for optimizing placement of these sensors.
